# 16-Channel biphasic current-mode programmable charge balanced neural stimulation

**DOI:** 10.1186/s12938-017-0385-0

**Published:** 2017-08-14

**Authors:** Xiaoran Li, Shunan Zhong, James Morizio

**Affiliations:** 1 School of Information and Electronics, Beijing Institute of Technology, Beijing, 100081 China; 2 Department of Electrical and Computer Engineering, Duke University, Durham, NC 27703 USA

**Keywords:** Neural stimulator, Electrical stimulation, Biphasic current, Constant current mode, Charge balance

## Abstract

**Background:**

Neural stimulation is an important method used to activate or inhibit action potentials of the neuronal anatomical targets found in the brain, central nerve and peripheral nerve. The neural stimulator system produces biphasic pulses that deliver balanced charge into tissue from single or multichannel electrodes. The timing and amplitude of these biphasic pulses are precisely controlled by the neural stimulator software or imbedded algorithms. Amplitude mismatch between the anodic current and cathodic current of the biphasic pulse will cause permanently damage for the neural tissues. The main goal of our circuit and layout design is to implement a 16-channel biphasic current mode programmable neural stimulator with calibration to minimize the current mismatch caused by inherent complementary metal oxide semiconductor (CMOS) manufacturing processes.

**Methods:**

This paper presents a 16-channel constant current mode neural stimulator chip. Each channel consists of a 7-bit controllable current DAC used as sink and source current driver. To reduce the LSB quantization error and the current mismatch, an automatic calibration circuit and flow diagram is presented in this paper. There are two modes of operation of the stimulator chip—namely, stimulation mode and calibration mode. The chip also includes a digital interface used to control the stimulator parameters and calibration levels specific for each individual channel.

**Results:**

This stimulator Application Specific Integrated Circuit (ASIC) is designed and fabricated in a 0.18 μm High-Voltage CMOS technology that allows for ±20 V power supply. The full-scale stimulation current was designed to be at 1 mA per channel. The output current was shown to be constant throughout the timing cycles over a wide range of electrode load impedances. The calibration circuit was also designed to reduce the effect of CMOS process variation of the P-channel metal oxide semiconductor (PMOS) and N-channel metal oxide semiconductor (NMOS) devices that will result in charge delivery to have less than 0.13% error.

**Conclusions:**

A 16-channel integrated biphasic neural stimulator chip with calibration is presented in this paper. The stimulator circuit design was simulated and the chip layout was completed. The chip layout was verified using design rules check (DRC) and layout versus schematic (LVS) design check using computer aided design (CAD) software. The test results we presented show constant current stimulation with charge balance error within 0.13% least-significant-bit (LSB). This LSB error was consistent throughout a variety stimulation patterns and electrode load impedances.

## Background

Neural integrated electronics continue to be designed and fabricated for a variety of scientific and biomedical applications and sensor technologies [1–7]. In vivo and in vitro electrophysiology research is becoming popular for neurological disorders as Parkinson’s disease, epilepsy, stroke, Alzheimer’s disease.

Neurological disorders occur in the central nervous system, the peripheral nervous system, and the autonomic nervous system. The main manifestation of the disease is in the feeling, movement, consciousness, and autonomic dysfunction. The etiology and pathogenesis of this disease are unclear. Many hypotheses such as neuronal signal pathway dysfunction, neuronal apoptosis and oxidative stress have attracted much attention. Neural stimulation is mainly used for central nervous system diseases, include neurodegenerative diseases (most common is Alzheimer’s disease, Parkinson’s disease, Huntington disease, and amyotrophic lateral sclerosis), vascular disease (also named stroke, include cerebral hemorrhage and cerebral infarction), functional disease (such as primary epilepsy), brain trauma, tumor, and infectious disease.

Electrophysiology equipment used for such medical applications use a close-loop neural recording and stimulation that is very capable of minimizing the effects of some of these neurological diseases. Implantable neural signal processing systems are comprised of neural recording, neural stimulators and radio transceiver. The neural recording subsection is used to acquire the neuronal signals and identify neural signatures during subsequent signal analysis. The neural recording system filters and amplifies multichannel electrode signals from a noisy environment. These analog signals are digitized using a data acquisition (DAQ) system and processed using real time software computing.

Implantable neural stimulators can be found in cochlear implants for the deaf, visual prostheses for blind, spinal cord stimulation for the paralyzed, muscle stimulation for a neural prosthetic, and deep brain stimulation for Parkinson’s disease [8]. A neural stimulator system provide charge balanced electrical current to multichannel electrodes implanted in tissue. This will create appropriate neuronal membrane potential to be excited and produce a corresponding signal response. Pulse frequency, amplitude, duty cycle and pulse shape of the biphasic current at the electrode is controlled by the stimulation parameters. The accuracy of the current pulse amplitude and pulse timing need to be quantified as an important part of the stimulation system specification.

**Fig. 1:**
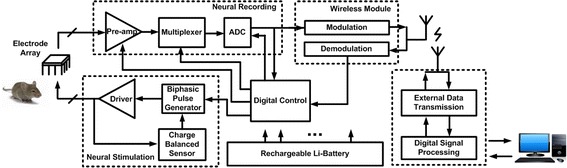
Block diagram of the neural signal processing. This block diagram contains neural recording path, neural stimulation path, digital control module, wireless transmission module, electrode array and battery

Figure 1 shows a block diagram of the neural interface that depict the recording and stimulation pathways. The stimulation path is used to deliver balanced charge into tissue, while the neural recording functions precondition the analog signals with amplifying, filtering and then analog-to-digital converting. Compared with the implementation of individual components, this integrated interface has the advantages of lower power, lower noise, smaller size, and higher precision.

Even though many methods have been published on how to maintain charge balance during neural stimulation [9–14], a charge imbalance still exists that will lead to permanent tissue damage. Passive charge balancing, active charge balancing and using the current source to remove residual charge have been previously described [15]. Power efficiency by energy recovery or recycling is also presented in many publications [14, 16, 8]. Although the design of Ref. [17] is power saving, the voltage-based stimulation has the disadvantage of a lack of control and the dynamic range is not wild.

Our objective with this proposed neural stimulator introduces calibration circuit and data flow to reduce charge imbalance to a least significant bit level caused by the effects of process variation and other non-ideal factors. Our design utilizes a high voltage process technology to allow for a wide output range needed for high impedance electrode technologies.

The remainder of this paper is organized as follows: “Methods” provides method, including system design, basic structure for neural stimulation, design specification, circuit implementation and layout design of the neural stimulation. “Results” presents the result of the proposed neural stimulation. “Discussion” discusses the results and the performance of the system. “Conclusion” presents the conclusion of this paper.

## Methods

This section will present an overview of the system design, the basic structure, the design specification and circuit implementation of the driver, calibration and the digital interface.

### System design

**Fig. 2:**
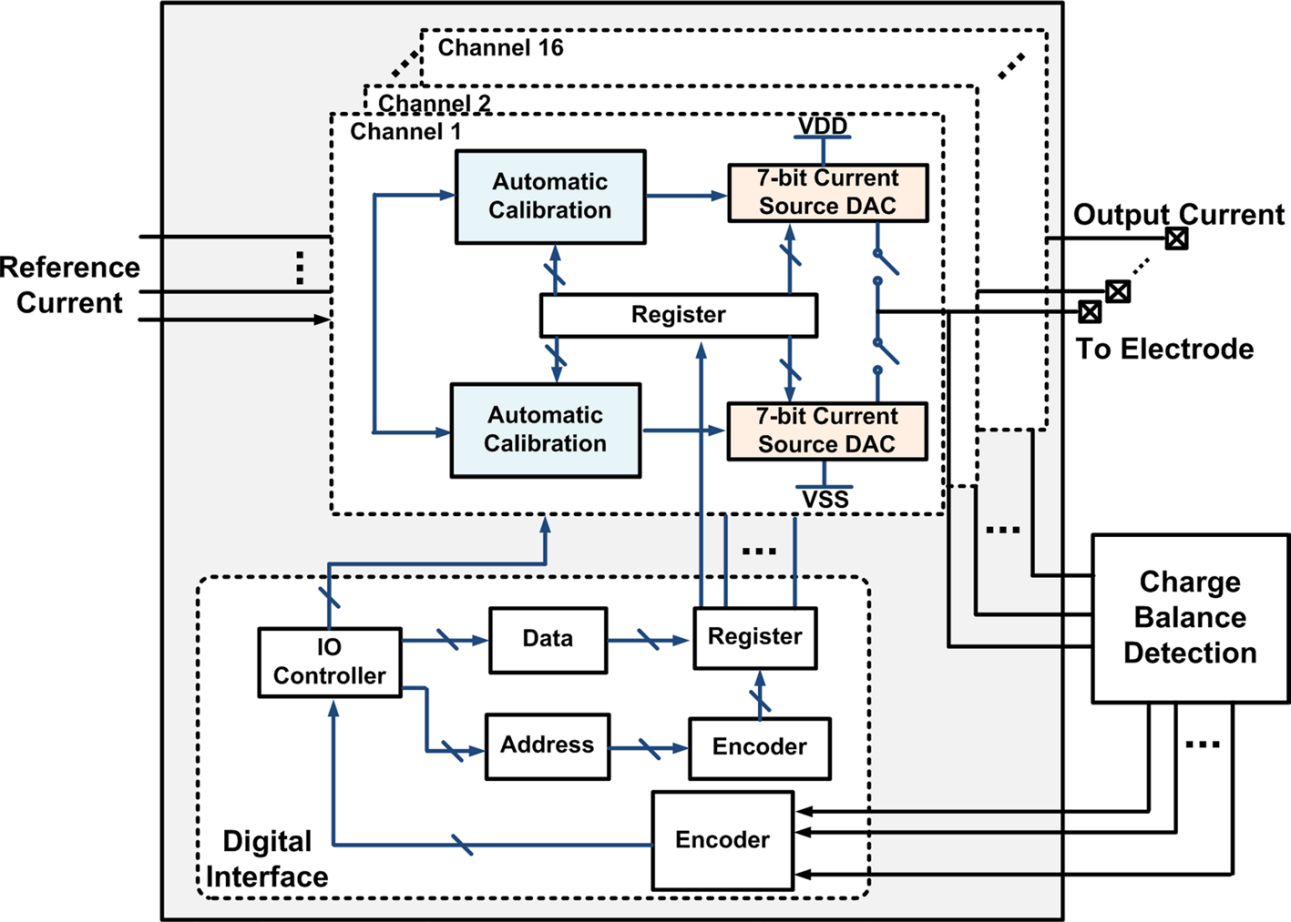
Block diagram of the system. The neural stimulator contains 16 channels. Each channel comprises a 7-bit current driver, polarity switches, calibration module and a register. The digital interface provides the control signal

A block diagram of the 16-channel neural stimulator is shown in Fig. 2. Each channel comprises a 7-bit current driver, polarity switches, and a 10-bit d-latch register to store the calibrated current level per channel. To reduce the least-significant-bit (LSB) quantization error and the current mismatch, an automatic calibration flow diagram is presented in this paper. The digital interface is used to control current driver switches and calibration switches all of the channels.

### Basic structure for neural stimulation

**Fig. 3:**
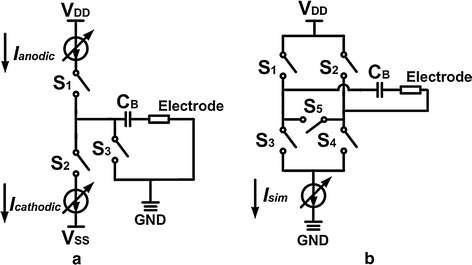
Conventional stimulator configuration. Illustrations of **a** dual supplies stimulator, and **b** single supply stimulator

There are several types of current mode stimulators. The single current mirror stack design approach is shown in Fig. 3a that depicts a positive currents source tied to V_DD_ and an negative currents source tied to V_SS_. Figure 3b shows a similar approach but with dual current mirror stacks in the current driver [11, 12, 18, 19].

The power supply of the current driver shown in Fig. 3a, uses a bipolar power supply of V_DD_ and V_SS_, which have the opposite and equal polarity thus providing both source current and sink current respectively. The *I*_*anodic*_ and *I*_*cathodic*_ currents are programmable via the 7-bit register value to represent the anodic current and cathodic current, each of which are controlled by the switches S_1_ and S_2_. In Fig. 3b, there is a unipolar power supply of V_DD_ and GND to current driver circuit. In one phase, S_1_ and S_4_ turn on, S_2_ and S_3_ turn off, and the charge is delivered from the left side to the right side of the electrode. In the following phase, S_1_ and S_4_ turn off, S_2_ and S_3_ turn on, and *I*_*sim*_ flows from the right side to the left side of the electrode.

**Fig. 4:**
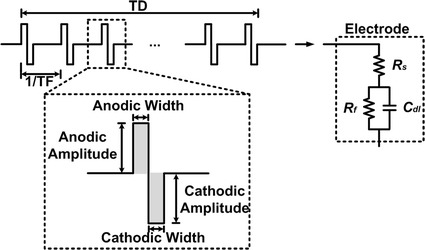
Biphasic current stimulator model. Illustrate of the biphasic current stimulator pulse and the simplified electrode model. An anodic pulse is followed by an equal cathodic pulse with an opposite polarity charge. TF (train frequency) is used to define the time period between each biphasic current pulse. TD (time duration) represents how long the interval pulse will last

The model of the biphasic current stimulator pulse and the typical electrode model are shown in Fig. 4, using both current source and sink [10, 14, 20]. An anodic pulse injects charge from the electrode into the neural tissue followed by an equal cathodic pulse with an opposite polarity charge in order to maintain the charge balance. TF is the train frequency, which is used to define the time period between each biphasic current pulse. TD is time duration, which represents how long the interval pulse will last.

Many researches have been proposed a variety of micro-electrodes models [21, 22]. The model of the electrode we used is found in the right side of the diagram in Fig. 4. This model electrode contains a parallel resistor *R*_*f*_ and capacitor *C*_*dl*_ with and a series resistor, *R*_*s*_. *R*_*s*_ is the solution spreading resistance and *C*_*dl*_ is the double-layer capacitance, while *R*_*f*_ is the faradaic resistance [10].

### Design specifications

The basic specifications of stimulator system must have several key characteristics including low power consumption, small physical size, long lifetime and reliability, high channel count of electrodes, and only a few external components [17]. For implantable neural stimulation, the procedure of implantation may cause mechanical damage. Small physical size will reduce tissue damage during implantation [15]. Once implanted, longer lifetime and higher reliability mean reducing the number of replacements, thereby reducing the possibility of damage during the implantation process and the user’s economic losses. Also we have more challenging requirements as constant current charge balance and high voltage range across a wide range of load impedances.

Neural stimulation is used to apply charge to neural tissue from the electrode [17]. The membrane potential of neurons is externally excited, and the neuron fires or action potential or spike as a response. Many factors, such as circuitry and frequency of stimulation, the shape of the waveform, and the location of the electrode, may affect the charge duration and transfer requirements of the stimulation pulses [23].

The impedance of the microelectrode is an important consideration. Many other electrode models are used with some form of conductance or a constant-phase element [8, 16, 17]. Each channel of the stimulator will drive high impedance electrodes load, which may vary in a wide range. The stimulation charge to neural tissue needs to be in the microampere to milliampere current range. The current is equal to the voltage divided by the load resistance. Since the high-voltage process will charge the maximum stimulation current into high impedance electrodes, the high voltage process is applied in this paper.

Another important consideration is that of safety. Several methods have been chosen to achieve this goal. Because prolonged DC current and charge accumulation in the neural tissue causes permanent damage. The constant current driver needs to maintain charge balance. To provide charge balanced stimulation, the accuracy and linearity of neural stimulation is important. In this paper, we propose an automatic calibration method to maintain an accurate charge balance output across the best case and worst case models of the complementary metal oxide semiconductor (CMOS) device manufacturing process variations [24].

### Circuit implementation

**Fig. 5:**
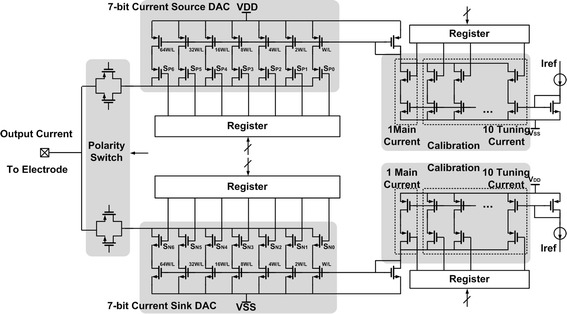
Schematic of one-channel neural stimulator. Illustrate the detail of one channel design, which consists of a 7-bit current digital-to-analog converter (DAC), an automatic calibration module, and the digital control interface

We developed a 16-channel biphasic current mode stimulator. Each unit consists of a 7-bit current Digital-to-Analog Converter (DAC), an automatic calibration module, and a digital control interface, which is shown in Fig. 5. The stimulator can be used in stimulation mode or calibration mode.

#### Current driver

**Fig. 6:**
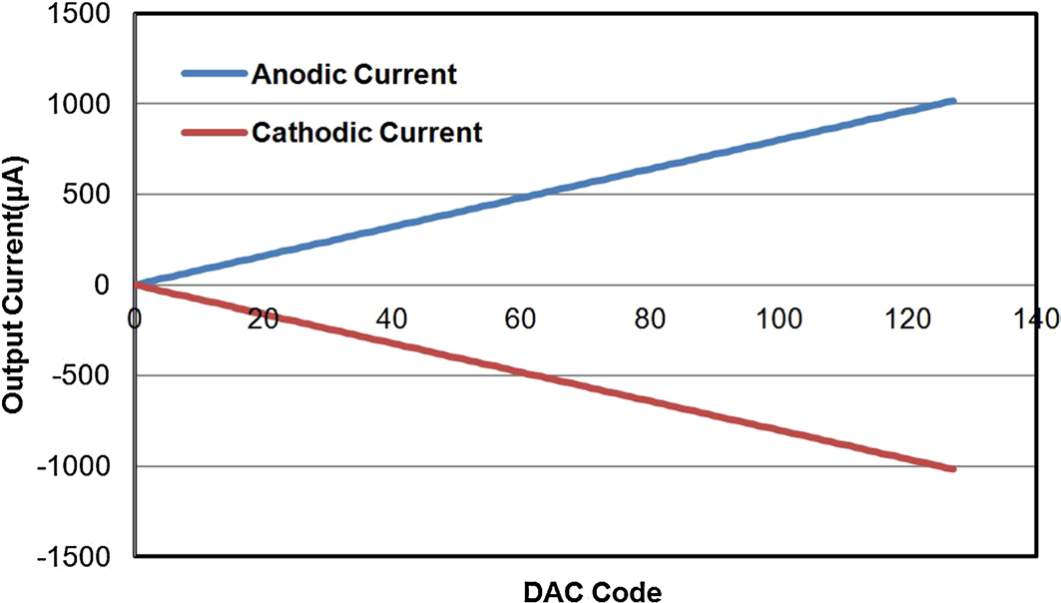
Step simulation waveform. The simulation result between output stimulation current and input DAC code of anodic current and cathodic current. The code is from 0 to 127

The schematic of each channel is shown in Fig. 5. The 7-bit current DAC consists of the 7-bit binary-weighted current sink DAC and the current source DAC. The digital register controls the transmission gates to choose which branch is on. The sink and source currents are controlled by a polarity switch. Figure 6 shows the cathodic and anodic currents of the current driver, according to the DAC code from 0 to 127. Both currents can be mirrored from 8 to 512 μA with a LSB current of 8 μA, so both current stimulations can be measured from 0 to 1016 μA.

#### Calibration

To maintain charge balance during electrical stimulation and reduce the effect of CMOS process variations, temperature, and the power supply, an automatic calibration technique is proposed in this design. One main current and ten tuning currents form the current input to the DAC. The default setting is to turn five tuning currents on while the other five currents are off. When the anodic and cathodic currents are unequal, the calibration will start automatically.

**Fig. 7:**
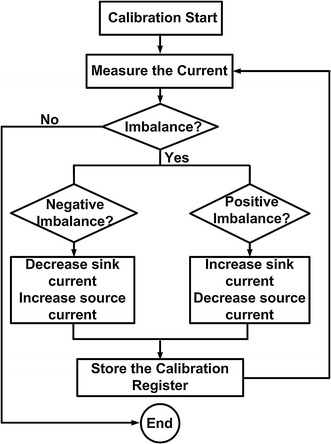
Flow chart of calibration process. Illustrate the calibration process when the calibration mode is on

When the stimulation mode is on, the calibration will be set as a default code and start to deliver charge. When the calibration mode is on, the voltage of the output node will be monitored by the off-chip analog-to-digital converter (ADC) and will send a signal back to the digital interface, which will send the control signal to the calibration register. A flow chart of the calibration process is shown in Fig. 7.

When the calibration starts, the maximum current is measured firstly. If the current has a negative imbalance, then the calibration register should be changed to increase the reference of the current source DAC or decrease the reference of the current sink DAC. If the current has a positive imbalance, then the reference of the current sink DAC should be increased or the reference of the current source DAC should be decreased. When the current is balanced, the calibration register stores the calibration data. A stable and charge-balanced current output can be achieved by adjusting the reference current.

#### Digital interface

The block diagram of the digital interface is shown in Fig. 2. Each channel of the stimulator drives high-impedance electrodes with individual channel controls for enable/disable, current, and phase. The current for each driver can be adjusted from 1 to 1016 μA using a 7-bit register. Because there are 16 channels, this register is 7 × 16 (112) bits wide. The current direction of each biphasic driver is controlled by another register.

The I/O controller controls the data and address paths. The data path is used to control the switches of the current drivers and the calibration, while the address path is used to choose each channel. The I/O also controls the transmission gate of each channel to switch the anodic and cathodic currents.

### Layout design

All individual circuit layouts for the constant current driver, calibration circuit layout and full chip layout was created to meet the design rules check (DRC) and layout versus schematic (LVS) rules of the 0.18 μm high voltage (HV) CMOS process technology using Mentor Graphics computer aided design (CAD) software.

The constant current driver layout was optimized to achieve the best device matching of the P-channel metal oxide semiconductor (PMOS) and N-channel metal oxide semiconductor (NMOS) devices using common centoid layout techniques. In addition, the minimum gate lengths of these devices were selected to be 2 or 3 times the minimum gate length of 0.18 μm to minimize the effect of channel length modulation on small gate lengths.

As with the constant current driver layout previously mentioned above, we used common centoid layout techniques of the calibration current mirrors to achieve the best device matching. Minimum gate lengths of these devices were also set to be 2 or 3 times the minimum gate length of 0.13 μm to minimize the effect of channel length modulation on small gate lengths.

**Fig. 8:**
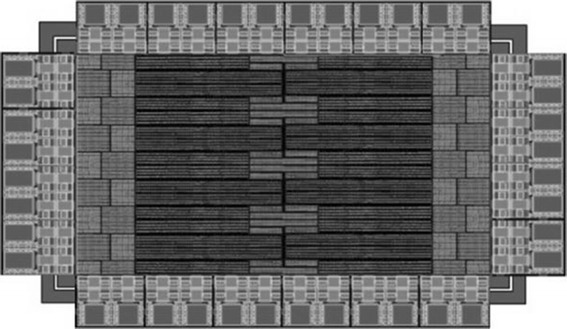
Screen capture of layout. Illustrate the 16-channel neural stimulator designed in 0.18 μm HV-CMOS technology

**Fig. 9:**
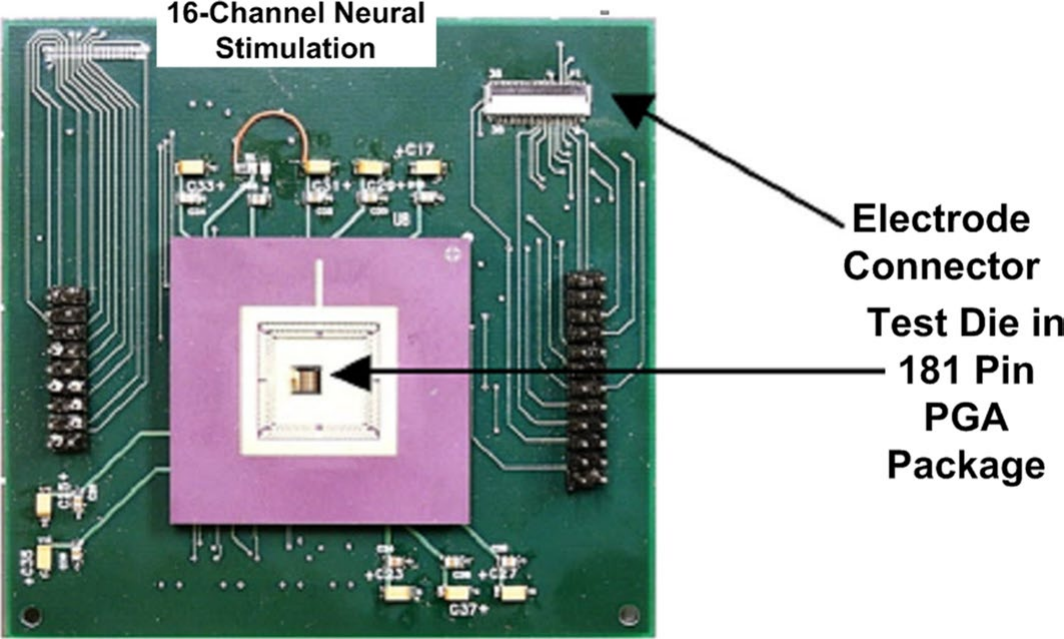
PCB test board. Illustrate the PCB test board of the chip. The chip is located in the center of the test board, while the electrode connector is located on the *upper right* of the test board

The full chip layout of the 16-channel stimulator chip was layout using 0.18 μm HV-CMOS technology, the layout and the printed circuit board (PCB) test board with a packaged application specific integrated circuit (ASIC) die is shown in Figs. 8 and 9, respectively. 16-channel stimulators are located common central. Guardring is applied to isolate noise and interference of each module. Electro-static discharge (ESD) protection is used on I/O PAD.

## Results

The power voltage supply was set to be ±20 V for tested as allowed by the HV-CMOS process specifications. The series load was varied from 1 to 20 KΩ to model the variation of stimulation electrode impedance of our simplified RC load model as shown in Fig. 4. Test results are summarized in Table 1.

**Table 1:** Performances of neural stimulation

Specification	Results
Technology	0.18 μm HV CMOS
Dynamic range	0–1016 μA
Voltage supply	±20 V
Stimulus mode	Current-mode
Mismatch percentage in entire stimulation current range	<0.13%
Channels	16

The current pulse was calculated by measuring the voltage across the resistor load. Hence, the maximum amplitude of the biphasic current pulses was achieved at the maximum voltage output of ±20 V. The maximum anodic and cathodic currents were 1015.63 and 1015.87 μA, respectively, with an 8-μA step size of the 7-bit amplitude resolution. Therefore, the current mismatch across the dynamic range of our output was below 0.3 μA.

**Fig. 10:**
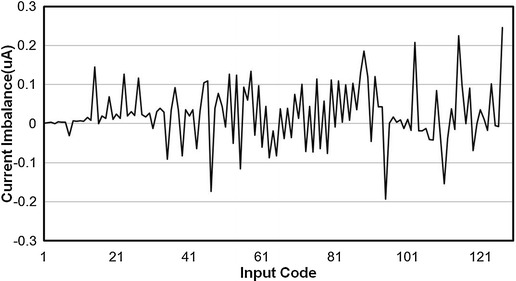
Current balancing performance. Illustrate the relationship between stimulation current imbalance and input DAC code of anodic current and cathodic current. The maximum stimulation current imbalance is 0.24 μA

**Fig. 11:**
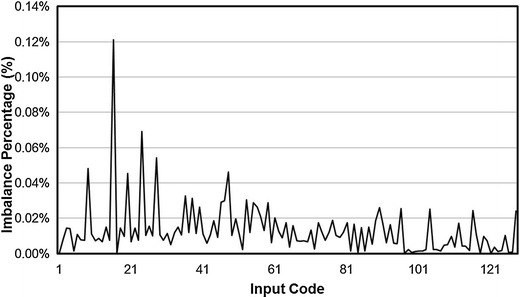
Current imbalance percentage. Illustrate the relationship between imbalance percentage error and input DAC code of anodic current and cathodic current. The charge delivery percentage errors are within 0.13% over the stimulation range

Charge balancing performance is measured by calculating the difference between anodic and cathodic currents; the ideal difference is zero. Figure 10 shows the current imbalance as a function of the DAC input code, while Fig. 11 shows the imbalance percentage as a function of the DAC input code. The worst current imbalance between anodic and cathodic current pulses occurred at the maximum stimulation current, while the imbalance percentage at the maximum stimulation current was less than 0.03%. Figure 11 shows that over the entire stimulation current range, the charge delivery percentage errors were less than 0.13%.

**Fig. 12:**
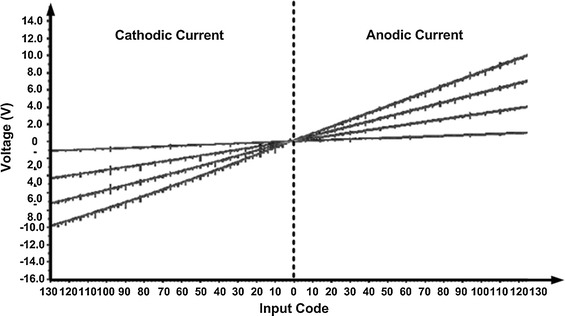
Load resister sweep result. Illustrate the relationship between output voltage and input DAC while the load is from 1 to 10 KΩ

The current driver was tested across a wide range of load resistances. Figure 12 shows the voltage of the output node as the resistor load was varied from 1 to 10 KΩ. The four curves represent the output voltage of four different resister loads. As shown in Fig. 12, the current remained constant even when the load resistance and corresponding output voltage changed.

## Discussion

We created a multi-channel biphasic constant current-mode neural stimulator with programmable current calibration. Each channel can operate in stimulation or calibration mode to ensure a minimal current mismatch so charge balance is achieved across the manufacturing processes. A 7-bit current DAC was used for each stimulation channel with small calibration current adjustments. Our stimulation system has a simplified architecture compared with other constant current driver designs with calibration in that we do not include any analog comparators or integrator circuits. Our approach simplifies the calibration result as a digital correction register value that is determined empirically and loaded during the calibration procedure, thus eliminating the need for any additional analog circuits as comparators and integrators.

We chose our constant current mode calibration stimulation approach for safety and precise control. Functional electrical stimulation is applied by injecting charge into neural tissue to detect the neural reaction [17]. There are two typical approaches for electrical stimulation. Voltage-based stimulation is designed to control the voltage between electrodes. This technique ensures higher energy efficiency, but it has the disadvantage of a lack of control, especially when the tissue impedance varies significantly. Current-based stimulation directly controls the current between electrodes. Although current-based stimulation suffers from inefficiency and higher power consumption, it remains used for its safety and increased control, as the charge delivered to the tissue is directly proportional to the current [6, 8, 25]. There are other approaches, such as switched capacitor charge delivery [26].

Automatic calibration was introduced in this design to reduce charge imbalance, since charge balance is an important design consideration in neural applications for safety reasons. A mismatched biphasic pulse will accumulate extra charge and induce DC current flow, which will permanently damage neural tissue. In reality, it is difficult to generate a matched current because of the mismatch of MOS and process variations. The power supply and temperature changes will also significantly affect the stimulation current. We used a charge balanced sensor and a return control signal to the chip for automatic tuning. For a stimulation current of 1 mA, a current mismatch less than 0.3 μA was produced.

A 7-bit current DAC was used to provide a high tuning resolution. As shown in Table 2, our stimulator maintains a charge mismatch error below 0.13%. These other calibration techniques [27–29] used on-chip integrators and comparators and redundant switches to reduce the anodic and cathodic charge mismatch. Our approach does not rely on any on-chip comparator or integrators since these components have inherent offset and tolerance when placed on the chip.

**Table 2:** Comparison of the proposed neural stimulation

Specification	[10]	[14]	[27]	[28]	This work
Technology	0.7 μm HV CMOS	0.18 μm HV CMOS	0.5 μm HV CMOS	0.35 μm HV CMOS	0.18 μm HV CMOS
Dynamic range (μA)	1000	504	3000	1000	1016
Voltage supply (V)	+6/−9	11.5	±8	±5	±20
Maximum mismatch current (μA)	4	N/A	2	0.3	0.24
Mismatch percentage in entire stimulation current range (%)	<0.4	<0.45	N/A	N/A	<0.13
Channels	16	8	N/A	N/A	16

## Conclusion

This paper presents a 16-channel integrated biphasic current mode neural stimulator chip with calibration. The stimulator circuit design was simulated and the chip layout was completed. The chip layout was verified using DRC and LVS design check using CAD software. Each channel can stimulate anodic and cathodic current, and the dynamic range reached 1 mA with 7-bit resolution. Calibration was used to minimize the current mismatch caused by the inherent CMOS manufacturing processes and other variations, and a charge imbalance below 0.13% was maintained. By designing the stimulator ASIC with high-voltage CMOS technology, the electrode load impedances can vary significantly while maintaining constant current stimulation. The proposed multi-channel current-mode stimulator can keep the charge balanced and work safely.

## Abbreviations

ADC: analog-to-digital converter
ASIC: application specific integrated circuit
CAD: computer aided design
CMOS: complementary metal oxide semiconductor
DAC: digital-to-analog converter
DRC: design rules check
HV: high voltage
I/O: input/output
LSB: least significant bit
LVS: layout versus schematic
NMOS: N-channel metal oxide semiconductor
PCB: printed circuit board
PMOS: P-channel metal oxide semiconductor
